# How Does the COVID-19 Pandemic Influence Histopathological Outcomes for Urologic Cancers?

**DOI:** 10.7759/cureus.26500

**Published:** 2022-07-01

**Authors:** Serdar Kalemci, Kasim Emre Ergun, Alp Akyol, Fuat Kizilay

**Affiliations:** 1 Urology, Ege University Faculty of Medicine, Izmir, TUR

**Keywords:** pandemic, bladder cancer, prostate cancer, urologic oncology, covid-19

## Abstract

Objective: The coronavirus disease 2019 (COVID-19) pandemic disrupted all routine health care services and resulted in a significant reconfiguration of urologic cancer services and care pathways across the globe. This study aimed to retrospectively determine the pandemic’s impact on the urologic oncological surgery outcomes at a high-volume referral center.

Materials and methods: We compared the number and histopathological outcomes of urologic oncological procedures in a referral center coded during the pandemic and data of the period before the pandemic as control. Data were extracted from patient files and hospital records. The pathological examination included a complete histopathological staging according to TNM stage.

Results: A total of 683 patients were included in the study, 424 (62%) of which were operated in the pre-pandemic period. There was a 39% decline in urologic oncological surgical activity in the pandemic, mostly in renal and prostate cancer. The mean tumor size was larger in renal cancer patients who underwent surgery during the pandemic (5.6 cm vs 4.5 cm, p=0.002). During the pandemic, more lymph node involvement was seen after radical cystectomy and prostatectomy (50% vs 27.8%, p=0.024 and 12.5% vs 4.5%, p=0.026, respectively). No differences in terms of main pathologic features were observed in patients undergoing radical orchiectomy.

Conclusion: COVID-19 appeared to adversely effect oncologic outcomes in patiens undergone surgery for prostate and bladder cancer. Tumor development induced by a delay in diagnosis may cause severe consequences for patients. Reprioritizion of non-deferrable urologic oncological seems crucial.

## Introduction

Coronavirus disease 2019 (COVID-19) is caused by a novel coronavirus, namely severe acute respiratory syndrome coronavirus 2 (SARS-CoV-2) [1]. Following the World Health Organization's (WHO) recognition of COVID-19 as a pandemic on March 11, 2020, COVID-19 has become a priority for the entire healthcare sector worldwide, including Turkey [2]. The COVID-19 pandemic has had a significant impact on cancer care and many other areas of health care. Urologists had to restructure their usual activities due to the global restrictions caused by the COVID-19 pandemic. 

Many scientific societies related to urological oncology have issued recommendations for the treatment of genitourinary cancers [3]. The major concern in this regard was the increase in cancer-related deaths and the decrease in survival due to delayed diagnosis and treatment of cancer patients. COVID-19 has been linked to poorer outcomes an increased likelihood of admission to critical care units, the need for invasive ventilation, and death in persons with cancer, according to early reports [4]. According to early data on urological oncology, it was underlined that urologic oncological activity was affected dramatically and these patients should be re-evaluated after the pandemic [5,6]. This study aimed to investigate how a delay during the COVID-19 pandemic in the diagnosis of urologic oncologic diseases has an impact on histopathological outcomes compared to the time before the COVID-19 pandemic.

## Materials and methods

A retrospective study was performed regarding patients who underwent urologic oncological surgery at Ege University Hospital, located in Izmir, Turkey, which is one of the leading university hospitals in the Aegean region with a patient capacity of 59 ward beds, including five level-1 intensive care unit beds. The daily practice of our department has also been changed compulsorily. At our institution, non-urgent cases were initially delayed then surgeries were delimitated. In March and April (at the start of the pandemic), only trauma, an acute obstruction that leads to renal failure, and selected oncology cases were evaluated for surgery with the suggestions of our hospital’s scientific committee and global urologic societies. After May, we started to operate other elective cases in a controlled manner. The study was exempted from approval of the Institutional Review Board due to its retrospective nature. All data analyzed were collected as part of routine diagnosis and treatment. The study was prepared in accordance with the ethical principles of the Declaration of Helsinki. In order to assess the impact of the COVID-19 on the histopathologic outcomes of the urologic oncological surgeries we used two time periods for comparison, Period 1 (from 1st of July 2018 till 31th of December 2019) and Period 2 (from 1st of July 2020 till 31th of December 2021).

Patient data were collected retrospectively from patient files and records. Data from patients who underwent radical orchiectomy, partial/radical nephrectomy, radical cystectomy, and radical prostatectomy were analyzed. Age of the patients, preoperative prostate-specific antigen (PSA) value, and histopathological data (pathological T stage, International Society of Urological Pathology (ISUP) grade group, positive surgical margin (PSM) and lymph node positivity) were recorded and evaluated. The pathological examination included a complete histopathological staging according to Tumor-Node-Metastasis (TNM) stage [7].

Data analysis was performed using SPSS software (version 24.0, IBM Corp., Armonk, NY, USA). Comparisons between two categorical variables were performed using Chi-Square or Fisher’s exact test and Mann-Whitney test was used for continuous variables with non-normal distribution and t-test was used for dependent groups showing normal distribution. A p-value <0.05 was considered statistically significant. 

## Results

A total of 683 patients were included in the study, 424 (62%) of which were operated in the pre-pandemic period. Compared to the pre-pandemic period, there was a 39% decrease in the number of urologic oncological surgical procedures. The decrease in surgeries was observed most prominently in renal cancer surgery with 50.8% and radical prostatectomy surgery with 43.4%. Renal cancer surgery was performed in 177 (67%) and 87 (33%) patients in pre-pandemic and pandemic periods, respectively. When the two periods were compared in terms of the mean age of the patients, the number of patients who underwent going nephron-sparing surgery, tumor side, tumor type and grade, and pathological tumor stage, there was no significant difference between the two groups (Table 1). The mean tumor size of patients who underwent surgery for renal cancer during the pandemic period was larger (5.6 cm vs 4.5 cm, p=0.002).

**Table 1:** Characteristics of patients undergoing kidney cancer surgery

Characteristic	Pre-pandemic n=177	Pandemic n=87	P value
Age, years	61.6 ± 13.5	58.9 ± 13	0.117
Tumor size, cm	4.5 ± 2.6	5.6 ± 2.9	0.002
Operation Type, n (%)			0.717
Partial	63 (35.6)	29 (33.3)	
Radical	114 (64.4)	58 (66.7)	
Side, n (%)			0.514
Right	93 (52.5)	42 (48.3)	
Left	84 (47.5)	45 (51.7)	
Tumor Type, n (%)			0.093
Clear cell	120 (67.8)	52 (59.8)	
Papillary	17 (9.6)	13 (14.9)	
Chromofobe	21 (11.9)	11 (12.6)	
Oncocytoma	15 (8.5)	4 (4.6)	
Other	4 (2.3)	7 (8)	
Tumor Grade, n (%)			0.340
Grade 1	10 (8.3)	1 (2)	
Grade 2	67 (55.8)	26 (52)	
Grade 3	34 (28.4)	19 (38)	
Grade 4	9 (7.5)	4 (8)	
Stage, n (%)			0.284
1	106 (67.5)	48 (57.8)	
2	10 (6.4)	7 (8.4)	
3	41 (26.1)	28 (33)	

A total of 310 patients underwent radical prostatectomy. The mean age of patients who underwent radical prostatectomy during the pandemic was lower (63.3 vs 65.8, p=0.001). During the pandemic, it was seen that robot-assisted laparoscopic radical prostatectomy was preferred as the surgical treatment compared to the previous period (59.8% vs 46%, p=0.019). There was no significant difference between the two groups in terms of PSA values, ISUP grade, surgical margin positivity rate, and pathological stage (Table 2). During the pandemic, men were likely to be diagnosed at a more advanced stage (pN1(+) patients) than those diagnosed during the same period before the pandemic (12.5% vs 4.5%, p=0.026).

**Table 2:** Characteristics of patients undergoing radical prostatectomy (PSA: Prostate-specific antigen, ISUP: International Society of Urological Pathology, LN: Lymph node)

Characteristic	Pre-pandemic n=198	Pandemic n=112	P value
Age, years	65.8 ± 6	63.3 ± 6.1	0.001
PSA, ng/mL	11.2 ± 11.7	9.15 ± 8.9	0.109
ISUP Grade, n (%)			0.100
1	26 (13.1)	11 (9.9)	
2	73 (36.9)	56 (50)	
3	47 (23.7)	27 (24.1)	
4	36 (18.2)	12 (10.7)	
5	16 (8.1)	6 (5.4)	
Operation Type, n (%)			0.019
Open	107 (54)	45 (40.2)	
Robot-Assisted	91 (46)	67 (59.8)	
Surgical Margin, n (%)			0.654
Negative	133 (67.2)	78 (69.6)	
Positive	65 (32.8)	34 (30.4)	
LN Metastasis, n (%)			0.026
Negative	189 (95.5	98 (87.5)	
Positive	9 (4.5)	14 (12.5)	
Stage, n (%)			0.070
pT2	98 (49.5)	69 (61.6)	
pT3a	72 (36.4)	27 (24.1)	
pT3b	28 (14.1)	16 (14.3)	

We performed significantly more radical cystectomies during the pandemic (32 (64%) vs 18 (36%), p=0.033). A trend toward a higher rate of lymph node involvement and mean tumor size are notable during the pandemic period (50% vs 27.8%, p=0.024, 4.7 vs 3.5, p=0.037). However, no difference in terms of mean age of patients, rate of variant histology, and pathological tumor stage were found (Table 3). The rate of patients with the pT3 stage was higher in patients who were operated on during the pandemic, but this difference was not significant (p=0.353). Overall, 31 patients underwent radical orchiectomy for testicular cancer in the pre-pandemic period and 28 patients in the pandemic. There was no difference between the two periods in terms of mean age of patients, mean tumor size, serum tumor marker levels, tumor type, and stage of the disease.

**Table 3:** Characteristics of patients undergoing radical cystectomy (LN: Lymph node)

Characteristic	Pre-pandemic n=18	Pandemic n=32	P value
Age, years	66.3 ± 5.9	66.2 ± 7.7	0.936
Tumor Size, cm	3.53 ± 1.42	4.7 ± 2.0	0.037
Variant Histology, n (%)			0.700
Negative	12 (66.7)	23 (71.9)	
Positive	6 (33.3)	9 (28.1)	
LN Metastasis, n (%)			0.024
Negative	13 (72.2)	16 (50)	
Positive	5 (27.8)	16 (50)	
Stage, n (%)			0.353
pT2	8 (44.4)	12 (37.5)	
pT3	7 (38.9)	18 (56.3)	
pT4	3 (16.7)	2 (6.3)	

## Discussion

During the pandemic, Izmir, which is located in the west of the country, has been one of the cities most affected as it is the third most populated city in the country. COVID-19 has caused major disruptions in cancer care, including prevention, diagnosis, and surgery. As in the rest of the world, patients in Turkey have had difficulties in being diagnosed with cancer, since many hospitals serve as pandemic hospitals. Furthermore, patients diagnosed with cancer experienced serious difficulties in reaching the necessary treatment due to the postponement or cancellation of their planned oncologic surgeries. A global survey during the early pandemic demonstrated that the clinical practice of 93% of urologists was affected and underlined the importance of post-pandemic evaluation of the impact of moderate cancellation of oncological surgeries [6]. In this study, we aimed to evaluate the impact of the pandemic on outcomes of urological oncological surgeries at a high-volume oncological referral center by comparing them with the surgeries in the pre-pandemic period.

Since the pandemic is an unprecedented and challenging situation for all countries in the world, health systems and urologic cancer services have had to deal with extra burdens, such as restricted theater availability for elective oncology. In a recent study, a 2-7% decrease in urological cancer surgeries in both public and private centers was observed respectively [8]. In another local study comparing the first year of the pandemic with the pre-pandemic in a high-volume center in Milan, Italy, a significant decrease was found in the number of urological oncological surgeries, similar to our study, and also they demonstrated that this reduction was largely due to radical prostatectomy surgery [9]. This decrease in the number of patients receiving prostate cancer treatment can be explained by the difficulties in reaching the urology outpatient clinics. In addition, the fact that patients generally prefer private sector hospitals that do not serve as pandemic hospitals may have affected these differences. At the beginning of the pandemic, the guidelines stated that prostate cancer treatment could be delayed [10]. However, in high-risk patients, treatment delay is associated with poor oncologic outcomes and if there is to be a delay, it is emphasized that this period should not exceed 12 months [11]. In our study containing approximately two years in the pandemic, there was no difference between the two periods in terms of the pathological stage however lymph node positivity was higher than in the pre-pandemic period. Although a similar rate of locally advanced disease was detected between both periods, this difference in lymph node involvement rates may be due to the surgeon's choice of lymph node dissection margin. In a large study using the national cancer database as a guide for urologists in the pandemic, it was stated that delaying prostate cancer treatment (not more than 12 months) does not lead to negative oncological results [12]. Moreover, it has been suggested that neoadjuvant hormonal therapy may be considered for high-risk patients [13].

In our study, similar to prostate cancer, there was a decrease in the number of renal cancer surgeries during the pandemic period, and the mean tumor size of patients who underwent surgical treatment for renal cancer during the pandemic increased compared to the previous period but this increase did not reflect negatively on the pathological outcomes of the patients. In a review on this subject, it has been suggested that the surgery of cT1 patients, who can be followed up under normal circumstances, may be delayed for a while, but cT2 patients should be considered as a candidate for surgery, particularly if they have unfavorable preoperative characteristics [13]. A multicenter study on small renal mass growth kinetics showed that none of the patients developed metastatic disease during follow-up, but it was underlined that biopsy from the mass, if possible, could be a guide in this regard, since the course of some masses may be different [14].

Muscle-invasive bladder cancer surgery differs from prostate and kidney cancer in terms of delaying treatment. The European Association of Urology (EAU) recommends offering radical cystectomy in T2-T4a N0M0 patients within three months [3]. In patients not receiving neoadjuvant chemotherapy, more than 12 weeks between diagnosis of muscle invasion and cystectomy has been shown to be associated with worse outcomes [15]. In fact, similar worse outcomes occurred when the surgeries of patients who received neoadjuvant chemotherapy were delayed for more than 10 weeks [16]. Moreover, It does not seem feasible for patients to receive neoadjuvant chemotherapy treatment during the pandemic, when it is already difficult to reach standard health care services during COVID-19. As a result, radical cystectomy should be regarded as a deferred emergency rather than an elective procedure. In our study, none of our patients received neoadjuvant chemotherapy. Similar to renal cancers, an increase in mean tumor size was observed, probably due to delays, in addition, more lymph node involvement was found in patients who underwent radical cystectomy during the pandemic.

Testicular cancers are also distinguished from other urological cancers by usually presenting as a unilateral scrotal testicular mass detected by the patient. It has been shown that a delay lasting four to six months may increase the probability of metastatic disease in testicular cancer [17]. Active surveillance is an option even in high-risk patients with stage 1 disease [18]. However, in patients with stage 2 disease, retroperitoneal lymph node dissection and chemotherapy options should be considered as adjuvant treatments and a final decision should be made after considering the side effects of both treatments. In summary, testicular cancer should be treated in a timely manner due to its curative nature [13].

This study is limited by its retrospective nature and single-center experience. Another limitation is that we did not include patients with transurethral bladder tumor resection, since we only included radical urologic oncologic surgeries. However, we provided results of the major urologic oncological surgeries in a high-volume referral center in the country's third-largest city, which has been severely affected by COVID-19.

## Conclusions

COVID-19 has significantly altered the management of urological cancer care and practice. Patients with urological cancer and urologist face various challenges during this period. Our study clearly showed a 39% decrease in the number of urologic oncological surgical procedures, notably in renal and prostate cancer surgery during the pandemic and the mean tumor size was larger in patients undergoing surgery for renal and bladder cancer. Another and most important finding was that rate of lymph node involvement, which will significantly affect the survival of patients was higher in patients who underwent surgery for prostate and bladder cancer during the pandemic. Future studies are needed to evaluate the impact of COVID-19 on long-term outcomes.
